# Determining the optimal dosimetric leaf gap setting for rounded leaf‐end multileaf collimator systems by simple test fields

**DOI:** 10.1120/jacmp.v16i4.5321

**Published:** 2015-07-08

**Authors:** Weiguang Yao, Jonathan B. Farr

**Affiliations:** ^1^ Department of Radiation Oncology St. Jude Children's Research Hospital Memphis TN USA

**Keywords:** Optimization of dosimetric leaf gap, tongue and groove effect, individual QA

## Abstract

Individual QA for IMRT/VMAT plans is required by protocols. Sometimes plans cannot pass the institute's QA criteria. For the Eclipse treatment planning system (TPS) with rounded leaf‐end multileaf collimator (MLC), one practical way to improve the agreement of planned and delivered doses is to tune the value of dosimetric leaf gap (DLG) in the TPS from the measured DLG. We propose that this step may be necessary due to the complexity of the MLC system, including dosimetry of small fields and the tongue‐and‐groove (T&G) effects, and report our use of test fields to obtain linac‐specific optimal DLGs in TPSs. More than 20 original patient plans were reoptimized with the linac‐specific optimal DLG value. We examined the distribution of gaps and T&G extensions in typical patient plans and the effect of using the optimal DLG on the distribution. The QA pass rate of patient plans using the optimal DLG was investigated. The dose‐volume histograms (DVHs) of targets and organs at risk were checked. We tested three MLC systems (Varian millennium 120 MLC, high‐definition 120 MLC, and Siemens 160 MLC) installed in four Varian linear accelerators (linacs) (TrueBEAM STx, Trilogy, Clinac 2300 iX, and Clinac 21 EX) and 1 Siemens linac (Artiste). With an optimal DLG, the individual QA for all those patient plans passed the institute's criteria (95% in DTA test or gamma test with 3%/3 mm/10%), even though most of these plans had failed to pass QA when using original DLGs optimized from typical patient plans or from the optimization process (automodeler) of Pinnacle TPS. Using either our optimal DLG or one optimized from typical patient plans or from the Pinnacle optimization process yielded similar DVHs.

PACS number: 87.55Qr

## I. INTRODUCTION

A multileaf collimator (MLC) is a sophisticated system with complex mechanical and dosimetric properties. Individual patient quality assurance (QA) is required for MLC‐involved intensity‐modulated plans (i.e., intensity‐modulated radiation therapy (IMRT) and volumetric‐modulated arc therapy (VMAT)) to verify agreement between the planned and delivered doses, which is critical for patient care.^(1)^ Over two decades, MLC properties, their uncertainties, and their potential clinical effects have been extensively investigated.^2^, ^3^, ^4^, ^5^, ^6^, ^7^, ^8^, ^9^, ^10^, ^11^, ^12^ With recently developed narrow leaf MLC systems and their applications in hypofractionated treatment, it is necessary to more accurately determine the properties and associated uncertainties. Narrow leaf MLC systems, such as the Varian 120 high‐definition (HD) MLC, Siemens 160 MLC, and Elekta160‐leaf Agility MLC, have larger interleaf leakage compared with wide leaf MLCs. Sometimes, however, an optimal value is also desired, besides the one accurately measured. This is because there exist deviations (e.g., irregularly shaped fields) or parameters that were ignored or not fully modeled (e.g., the spatial distribution of MLC transmission and the tongue‐and‐groove effects) in the dose calculation algorithm. Additionally, there could be related quantities that are difficult to accurately measure (e.g., output factors of small fields due to volume effect of the ion chamber) or uncontrollable factors (e.g., target and organ movement) during dose delivery. Use of an optimal value minimizes the effects of these uncertainties.

For rounded leaf‐end MLC systems, the Varian Eclipse treatment planning system (TPS) requests that the user input the value of two dosimetric parameters of MLC during commissioning: transmission ratio and dosimetric leaf gap ((DLG), also called dosimetric leaf separation). The DLG is related to the gap between light and radiation fields, and can be measured by extrapolating the size of static or dynamic fields formed by MLC leaves to the size under which the measured dose equals the MLC leakage. However, due to the uncertainties, using the measured DLG in a dose calculation algorithm, such as the analytic anisotropic algorithm, often results in lower‐than‐expected agreement between the planned and delivered doses during the individual QA.^(13)^ To seek closer agreement, replanning, including smoothing the fluence, is sometime performed, but is a reluctant practice because it is time‐consuming.^(14)^ Furthermore, the new plan may still not pass the QA, and smoothing the fluence often decreases the plan quality. Another — perhaps necessary — way to improve the agreement is to tune the value of the DLG, for some typical patient plans, to obtain the optimal one.^(13)^ Here, we interpret the optimal value as that suitable for as many plans as possible delivered by a certain linac. In this sense, typical patient plans may not be the best reference for the optimal DLG if one wants the optimal DLG to work for nontypical plans, as well. It has been reported that higher failure rate happens at sites with either very large or very small target volumes surrounded by sensitive organs.^(15)^

Small fields formed by MLC leaves are common in IMRT/VMAT plans, but the dosimetry of small fields has been one of the biggest challenges in radiation therapy, especially for irregularly shaped fields, which are common in IMRT/VMAT plans.^16^, ^17^ The dose measurement of small fields is subject to the ion chamber volume effect, stem effect, leaking, and other effects, and the analytical dose calculation algorithm for small fields is subject to the lateral electron nonequilibrium and penumbra, in addition to those inputs from measurements. Monte Carlo simulation for small fields can be accurate, but very slow. Furthermore, the T&G effects can significantly change the dose distribution, especially when the dose distribution is verified field‐by‐field, as done in individual IMRT QA.^(5)^ Compared to the fields formed by jaws or cones, the fields formed by MLCs have specific penumbras. The penumbra in the lateral direction depends on the structure of the rounded leaf‐end, and the penumbra in the longitudinal direction relies on the structure of tongue and groove. Traditional measurements for rounded leaf‐end effects (virtual gap) use various lateral field sizes, but a fixed longitudinal field size (i.e., 10 cm in the longitudinal direction). The lateral penumbra is accounted for by the measured DLG, but the longitudinal penumbra has a negligible effect on this DLG because of the large field size in this direction. However, actual IMRT/VMAT patient plans usually have segments with small longitudinal field sizes. Tuning the value of the DLG could compensate not only for the dose from the leaf gap to the longitudinal penumbra, but also for the dosimetry inaccuracy of small fields (gaps). Clearly, of the two dosimetric data inputs in the Eclipse TPS, transmission ratio affects dose in the entire jaw‐formed field and more uniformly than DLG does, whereas DLG mainly affects the dose from photons passing through the apertures. Thus, these two parameters have different functions dosimetrically. If the planned dose is consistently higher or lower than the measured dose, the transmission ratio should be checked first. The transmission ratio is easier to measure than DLG, but cannot effectively compensate the penumbras. Given these considerations, we introduced a wide range of T&G effects into fields commonly used in DLG measurements. These fields will be called test fields. The advantages of test fields over typical patient plans include a wider range of T&G extensions and leaf gaps, as well as independence from the MLC system, treatment site, and patient plan. Thus, test fields can provide a more complete and efficient way to determine the optimal DLG, which accounts for both the lateral and longitudinal penumbras. In order to specify the T&G effect on penumbras, we use the term T&G extension to differentiate the T&G effect due to interleaf leakage. We use the measured transmission ratio to search for the optimal DLG value so that the planned dose best agrees with the measured dose.

In this paper, we report our work on determining optimal DLG values by utilizing test fields for the Varian millennium 120 MLC (installed in Trilogy, 2300 iX, and 2100 EX), HD 120 MLC (installed in TrueBEAM STx), and the Siemens 160 MLC (installed in Artiste) in the Eclipse analytic anisotropic algorithm (version 10 and 11), and compare the results with those from the DLGs originally optimized from patient plans (Varian MLCs) and from the optimization process (automodeler) of Pinnacle TPS (Siemens MLC). The Varian MLC systems were used in sliding window mode, and the Siemens MLC system was used in step‐and‐shoot mode.

## II. MATERIALS AND METHODS

Before introducing our scheme, we emphasize that our linacs (TrueBEAM STx, Trilogy, Clinac 2300iX, EX2100 (Varian Medical Systems, Palo Alto, CA), and Artiste (Siemens Medical Solutions, Malvern, PA)) and Eclipse treatment planning system (TPS) (Varian Medical Systems) had been properly commissioned and maintained. This is important because optimization on a poorly commissioned system could mask some problems, and thus such a kind of optimization is meaningless. We followed AAPM Task Groups (TGs 40, 45, 51, 53, 114, and others) to perform commissioning and quality assurance. Particularly for IMRT, we participated in various RTOG clinic trials to confirm that our systems were properly commissioned and maintained.

Furthermore, the IMRT/VMAT individual QA process was strictly followed. All types of setup, such as the QA equipment (MapCHECK 2 and ArcCHECK; Sun Nuclear Corporation, Melbourne, FL), couch and gantry positions were double checked. The daily fluctuation of output factor was corrected by performing the dose calibration function of the SNC Patient 6.2 (Sun Nuclear Corporation).

Individual QA plans were generated by the TPS with the same version as that for planning. For IMRT plans, the gantry angle in the QA plans was always set to 0° so that the couch structure would not affect the delivered dose. For VMAT plans, the couch structure was included in planning, and before VMAT QA, the couch position was double checked.

### A. Test fields

**Figure 1:**
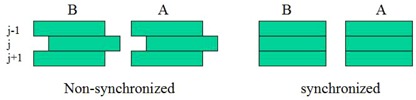
Test fields with nonuniformly (left pair) or uniformly (right pair) extended MLC leaves. “A” and “B” indicate the left and right carriages. During dose delivery, the leaves move at the same speed and in the same direction, so the gaps (i.e., pattern) do not change. Six different gaps (5, 10, 15, 20, 25, 30 mm) and five different extensions (0, 5, 10, 15, 20 mm) were used in this study; thus, there were 30 test fields.

DLG is often measured via extrapolating the lateral size of fields formed by uniformly extended MLC leaves to the size under which the measured dose equals the MLC leakage. In this situation, the T&G extension would be minimal. Practical IMRT and VMAT fields, however, are formed by nonuniformly extended MLC leaves, where the T&G extension becomes significant. To determine the optimal DLG for a wide range of T&G extensions and leaf gaps, we generated both uniformly and nonuniformly extended MLC‐formed test fields (see Fig. 1). During dose delivery, leaves move in the same direction and at the same speed, keeping the leaf‐formed pattern unchanged. For MLC systems used in sliding window mode, the test fields were constructed by using Varian MLC Shaper software, and for systems used in step‐and‐shoot mode, the test fields were generated via a field‐in‐field approach.

The gaps formed by opposite leaves in both uniformly and nonuniformly extended fields were 5, 10, 15, 20, 25, and 30 mm. Note that the minimal gap cannot be less than the manufacturer‐recommended limit, to prevent collision. For each gap, four nonuniformly extended fields had the difference of neighboring leaf extension (T&G) of 5, 10, 15, and 20 mm. Thus, there were 24 nonuniformly extended test fields and six uniformly extended test fields.

The field size, formed by the jaws, of each test field was $10\times 10\;{\text{cm}}^{2}$. In those test fields with sliding window technique, the window moved at a constant speed from left to right, and in those with step‐and‐shoot technique, a sufficiently large number of subfields (field‐in‐field) were uniformly distributed in the $10\times 10\;{\text{cm}}^{2}$ field. The minimum number of subfields was 12. With such a design, both planned and delivered doses must be uniformly distributed in the central region of the $10\times 10\;{\text{cm}}^{2}$ field. This approach can decrease uncertainty of the measured dose due to ion chamber positioning at the center of field.

Planned doses from test fields were calculated in the TPS with various values of DLG but a fixed number of monitor units (MUs); 400 MUs were used for each test field in our work. The voxel size in dose calculation was 2.5 mm in both lateral and anterior–posterior directions, and was defaulted to the CT slice thickness in the longitudinal direction. The planned dose at the center of each test field was compared to the measured dose. The optimal DLG was determined when the discrepancy (=100% (measured−planned)/planned) was minimized over the range of gaps and T&G extensions. To measure the delivered dose, we used Farmer‐type and PTW semiflex 31010 ionization chambers (ICs), which have collection volumes of $0.6\;{\text{cm}}^{3}$ and $0.125\;{\text{cm}}^{3}$, respectively (PTW, Freiburg GmbH, Germany). Because the dose in the center region of the test fields was designed to be uniform, the measured doses from Farmer‐type and semiflex ion chambers were almost the same. Note that with fixed MUs for each test field, the delivered dose is not related to the value of the DLG in the TPS. The delivered dose needs to be measured only once. The whole measurement takes typically within 1 hr, depending on the dose rate used.

### B. MLC systems

The optimal DLGs for three MLC systems were investigated: a Varian millennium 120 MLC, a Varian HD 120 MLC, and a Siemens 160 MLC. The millennium MLC has 40 leaf pairs with a projected width of 5 mm in the central region and 20 leaf pairs with a projected width of 10 mm in the outer region. They can form a maximum $40\times 40\;{\text{cm}}^{2}$ field. The HD MLC has 32 leaf pairs with a projected width of 2.5 mm in the central region and 28 leaf pairs with a projected width of 5 mm in the outer region. The maximum field formed by the HD MLC is $22\times 40\;{\text{cm}}^{2}$. The Varian MLC systems are designed with conventional T&G structure to decrease the interleaf leakage. The Siemens 160 MLC has a 5 mm leaf projection width to form a maximum $40\times 40\;{\text{cm}}^{2}$ field and has no T&G structure. To decrease the interleaf leakage, the Siemens system is installed in a linac with a tilt angle approximately 0.37° from the beam's eye view, resulting in a so‐called triangular T&G effect.^18^, ^19^ Because of the slight tilt, the shape of the “triangle” depends slightly on the location of the leaves in the field. This location dependence may increase the uncertainties and may cause a more complicated effect than the standard T&G effect.

### C. MLC transmission ratio

The transmission ratio is defined as the ratio of leakage and scatter dose in MLC leaves over the open field dose.^(2)^ The transmission is composed of inter‐ and intraleaf components. Because of the interleaf transmission, the transmission ratio under the interleaves is larger than that in any other region of the field. Particularly for the Siemens 160 MLC, the slightly tilted MLC results in unbalanced transmission ratio along the tilt direction.^(19)^ However, if the TPS uses a single value for transmission ratio, then this value should be the mean of the transmission ratio over a large field. In our work, we used a PTW 34001 Roos plane parallel ion chamber (PTW) and a Farmer‐type ion chamber to measure the transmission ratio in the central region of the fields. The plane parallel ion chamber has a 15 mm diameter collector and, thus, covers a projection of three leaves for 5 mm leaves and six leaves for 2.5 mm leaves. For the Farmer‐type ion chamber, the collector is 15.6 mm long and, thus, covers a similar number of leaves as the plane parallel ion chamber does when the Farmer‐type ion chamber is set to be perpendicular to the MLC leaves. We set the ion chambers at the center of the field, but also checked the interleaf transmission effect by setting the ion chambers at a half‐leaf projection width that was offset to the center of the field perpendicular to the direction of leaf motion. The measured transmission ratios were almost the same for both types of ion chambers and setups. In the TPS, we used the transmission ratios measured by the Farmer type ion chamber in solid water at $d_{\text{max}}$, with a source‐to‐surface distance of 100 cm and a $10\times 10\;{\text{cm}}^{2}$ field, although they were a little dependent upon depth due to the change of the photon energy spectrum.

### D. Dose agreement evaluation

A Sun Nuclear MapCHECK 2 was used to measure the delivered dose from the verification plan of the patient IMRT plan, and an ArcCHECK was used for VMAT plans. The SNC Patient 6.2 software was used to evaluate the agreement of planned and delivered absolute doses.

Our institute's QA criterion is 95% of measured point doses passing the DTA or gamma tests with 3%/3 mm and 10% threshold. For plans using the sliding window technique, the DTA test was performed. A dose comparison function Van Dyk Comparison (see the user manual of SNC Patient) was OFF for IMRT plans but ON for VMAT ones. These setups are commonly accepted for IMRT QA^(14)^ and agree with the 95% confidence analysis.^(1)^ The different choice of Van Dyk status in IMRT and VMAT QA might be attributed to the location of the detectors in the high‐ and low‐dose regions. For our IMRT QA setup with 5 cm solid water added onto the MapCHECK 2, the detectors are around the 80% PDD region, but for VMAT QA plans with the ArcCHECK, the detectors are most likely in the region <50% of target dose, especially for prostate plans. In low‐dose regions, the dose comparison (=100% (measured dose−planned dose)/planned dose) will be more sensitive to the planned dose than Van Dyk Comparison does, which calculates the difference against the planned dose at the normalization point, usually at the maximum dose in the plan (see the user manual of SNC Patient for more detail). Hence, when Van Dyk is ON, the QA pass rate will generally be improved. In the clinic, if the low‐dose region is less significant than the high‐dose region, then one may turn Van Dyk ON to obtain a more accurate QA pass rate in the high‐dose region. A more detailed analysis of Van Dyk effect on the QA pass rate is out of the scope of this work.

For IMRT plans using the step‐and‐shoot technique, we used the gamma test with 3%/3 mm, 10% threshold, and Van Dyk ON. Again, these settings are commonly accepted for the step‐and‐shoot technique, but we also investigated the dose agreement with Van Dyk OFF.

### E. Calculation of gap and T&G extension in patient plans

We used MLC files exported from Eclipse to analyze the T&G extension and gap distributions in patient IMRT plans using the sliding window technique. The gap was calculated as the distance between the leaf‐ends of the pair of leaves. To calculate the T&G extension, referring to Fig. 1, denote A(j), B(j) as the locations of the rounded leaf‐ends of the jth pair in carriage A and B respectively, and G(j)=A(j)−B(j) as the gap formed by the jth pair of leaves. The contribution of T&G extension from leaf B(j−1) to gap j was calculated as the exposed length of leaf B(j−1) to gap j:

(1) $$TGB(j-1,j)=\{\begin{array}{ll} 0 & if\;B(j-1)-B(j)<0,\\ G(j) & if\;B(j-1)-B(j)>G(j),\\ B(j-1)-B(j) & otherwise. \end{array}$$

For example, in the nonuniformly extended case of Fig. 1, TGB(j−1,j)=0 because the left leaf of the j−1th pair does not expose to the gap j, and TGB(j,j−1)=B(j)−B(j−1) because the part of left leaf of the jth pair is exposed to the gap j−1 and the exposed length equals B(j)−B(j−1).

The calculation of TGA(j−1,j) for carriage A can be done in the same way. The total T&G extension for gap j was taken as

(2) $$TG(j)=\frac{1}{2}[TGA(j-1,j)+TGA(j+1,j)+TGB(j-1,j)+TGB(j+1,j)].$$

In other words, in the calculation of T&G extensions, we did not separate tongue and groove, but treated them the same.

## III. RESULTS

### A. MLC transmission ratio

The measured MLC transmission ratios at 6 MV are listed in Table 1. The Varian MLC systems have larger transmission ratios than does the Siemens MLC system.

**Table 1:** Measured MLC transmission ratios (TRs) for three types of MLC systems installed in five linear accelerators. The other (TR, DLG) optimization schemes refer to the optimization from typical patient plans for the Varian 120 MLC systems and the Pinnacle optimization process for the Siemens 160 MLC system

*MLC System*	*Linac*	*MLC TR (%)*	*Optimal DLG From Test Fields (mm)*	*DLG From Extrapolation (mm)*	(*TR, DLG) From Other Optimization Schemes (%, mm)*
Millennium 120	Trilogy	1.4	2.3	1.6	(1.25, 2.5)
Millennium 120	Clinac 2300 iX	1.4	2.3	1.6	(1.25, 2.5)
Millennium 120	Clinac 21 EX	1.4	2.5	1.8	Not done
High definition 120	TrueBEAM STx	1.3	0.6	0.3	(1.3, 0.8)
Siemens 160	Artiste 1	0.26	0.3	0.2	(1.24, 0.4)

### B. Optimal DLG

**Figure 2:**
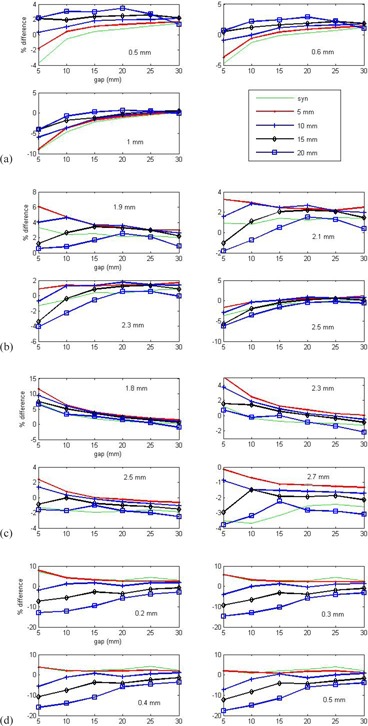
Percentage difference between measured and calculated doses of the test fields. The values of DLG used in the calculation are noted in the figure. The legend of curves gives the T&G extensions in the test fields, where “syn” is for synchronized MLC motion (i.e., no extension). A linac‐specific optimal DLG was selected as that with the minimal discrepancy in the investigated region of gaps and T&G extensions: (a) HD 120 MLC in TrueBEAM STx, (b) millennium 120 MLC in Trilogy, (c) millennium 120 MLC in Clinac 21 EX, and (d) Siemens 160 MLC in Artiste. The sliding window technique was used in (a)–(c), and the step‐and‐shoot technique was used in (d).

Figure 2 displays the percentage difference between measured and planned doses at 6 MV with various values of DLG in TrueBEAM STx, Trilogy, 21 EX, and Artiste. The result from our Clinac 2300 iX was very close to that from Trilogy. The following observations were made: a) no single value of DLG can result in a perfect dose match in these wide ranges of T&G extensions and gaps, but an optimal DLG can be selected to work in most of the regions; b) the percentage difference is very sensitive to the gap when the gap <10 mm for Varian millennium and HD 120 MLCs and <20 mm for Siemens 160 MLC, but a properly chosen DLG can decrease the sensitivity; c) as the gap increases, the dose agreement improves, and the dependence on T&G effect relatively decreases; d) some values of DLG work better in small T&G extension but worse in big T&G extension; e) as DLG increases, dose in plan will be higher than dose measured, and vice versa; and f) dose agreement is more sensitive to the value of DLG in Varian 120 MLC systems than in the Siemens160 MLC system.

Table 1 lists the optimal DLG determined from the test fields for the Eclipse TPS. As a reference, it also lists the DLG obtained from the uniformly extended test fields by extrapolating the delivered doses to be the same as the MLC transmission. The difference between the DLGs can be treated as a compensation of the small‐field dosimetry and T&G effects in the dose calculation algorithm. Interestingly, the optimal DLGs are consistently larger than the extrapolated ones. This information may be useful for improving the algorithm. The combination of transmission ratio and DLG, originally optimized from typical patient plans (those using the sliding window technique and VMAT in Eclipse) and the Pinnacle optimization process (those using the step‐and‐shoot technique), is also shown (Table 1). Although the MLC systems in Clinac 21 EX, Clinac 2300 iX, and Trilogy were all Millennium 120 MLC, the vendor told us that the MLC integrated with Clinac 21 EX was a little different from those in Clinac 2300 iX and Trilogy, but no details were given. This may explain the different DLGs in these three linacs.

### C. T&G extension and leaf gap distributions in typical patient plans

**Figure 3:**
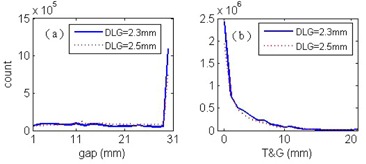
Distributions of leaf gaps (a) and T&G extensions (b) in a head‐and‐neck patient's IMRT plan in which the sliding window technique was delivered by using Trilogy.

Figure 3 displays the T&G extension and leaf gap distributions of an IMRT patient plan for the head and neck, with DLGs optimized from our test field (2.3 mm and the associated transmission ratio=1.6%), and from typical patient plans during commissioning (2.5 mm and the associated transmission ratio=1.4%), delivered by Trilogy with the sliding window technique. Both the patient plan and QA plan (verification plan) were generated by the same version of the TPS. The T&G extension was calculated by Eq. (2) and rounded to 1 mm, but the MATLAB software (MathWorks Inc., Natick, MA) interpolated the curves. Equation (2) was applied to all the control points. In Eclipse, the control point is used to control the MLC movement. In the figure, we combined the total counts of gaps ≥30 mm into the number of counts at gap=30 mm because, from Fig. 2, the dose agreement improves as gap increases and the difference will be less than 3% when the gap >30 mm. The gaps were distributed from 1 mm, with most being greater than 27 mm, and T&G extensions were distributed from 0 mm, with most being less than 3 mm (Fig. 3). Compared with these distributions, there were significantly more gaps >28 mm and T&G extensions <3 mm in the plan with a DLG of 2.3 mm than in that with a DLG of 2.5 mm. The results of test fields show that having a big gap and small T&G extension gives a high QA pass rate. Although the dose at each point is contributed to by many MLC segments, having more segments with large gaps and small T&G extensions increases the dose agreement.

### D. Validation of optimal DLG in patient plans

During our investigation of optimal DLG using the test fields, the dose distributions and individual QA pass rates from more than 20 patient plans were studied. The original plans used the combination of transmission ratio and DLG from either typical patient plans (those with sliding window technique and VMAT) or Pinnacle optimization results (those with step‐and‐shoot technique). Most of these original plans failed to pass the institute's QA criteria. However, with our optimal DLG that is linac‐specific, all the plans passed QA testing. After releasing our optimal DLG to the clinic no plan failed, although, theoretically, a single value of DLG will not work for all possible situations. In case a plan failed, one can use the result from test fields, such as in Fig. 2, to find the plan‐specific optimal DLG. For example, if the planned dose is higher than the measured dose in most of the region, a DLG value smaller than the linac‐specific value of DLG would improve the dose agreement.

1, 2, 3, 4 list the QA pass rate from three patients' IMRT QA plans. The first plan was for a large pelvic target, with the largest field being $29.3\times 30.5\;{\text{cm}}^{2}$, with 1091 control points and 851 MUs, for 200 cGy prescription dose per fraction, and normalized with 95% prescription dose to 100% PTV (the normalization way was the same for all three plans). The second plan was for a large head‐and‐neck target, with the biggest field being $30.6\times 21.2\;{\text{cm}}^{2}$, with 997 control points and 784 MUs, for 200 cGy prescription dose per fraction to the tumor. They were both delivered by the millennium 120 MLC using the sliding window technique. The third plan was for a right parietal target, with the biggest field being $18.2\times 10.5\;{\text{cm}}^{2}$, totally 146 segments and 484 MUs, for 183 cGy prescription dose per fraction to the tumor, and was delivered by the Siemens 160 MLC using the step‐and‐shoot technique. The first two plans were made in Eclipse with DLGs optimized from typical patient plans and our test fields. The third one was planned in Pinnacle with a transmission ratio of 1.24% and a DLG of 0.4 mm and in Eclipse with a transmission ratio of 0.26% and a DLG of 0.3 mm. For this plan, we checked the pass rate with the institute's QA criteria (3%/3 mm) and the pass rate with more tightened criteria (i.e., 2%/2 mm, 1%/1 mm, and Van Dyk OFF). Using an optimal DLG determined from the test fields generally improves the QA pass rate for all these criteria. The QAs failed to reach 95% for some fields planned with DLGs from the other optimization approaches, but passed with DLG from the test fields. Particularly for the third plan when Van Dyk was OFF, the QA pass rates were much improved when the transmission ratio and DLG were optimized from the test fields.

**Table 2:** Individual QA pass rate with different criteria for each field of a pelvis IMRT plan having the specified transmission ratio and DLG parameters, using the DTA test and the gamma test. The Van Dyk was OFF

*GA DTA*		*20*	*60*	*100*	*140*	*180*	*220*	*260*	*300*	*330*
TR=1.25%, DLG=2.5mm	3%/3 mm	97.5	96.5	98.8	96.8	96.5	96.8	97.4	94.1	96.5
	2%/2 mm	93.4	92.8	94.6	92.2	91.9	91.8	93.6	88.3	91.3
	1%/1 mm	82.0	80.4	86.5	80.9	79.2	79.0	84.0	77.2	81.1
TR=1.4%, DLG=2.3 mm	3%/3 mm	99.5	99.1	99.3	98	98.5	99.1	99.1	98.2	98.5
	2%/2 mm	98.4	96.5	97.8	95.1	95.0	95.7	95.8	93.0	95.6
	1%/1 mm	91.7	87.1	91.9	87.4	86.5	86.5	89.3	86.7	89.2
*GA Gamma*		*20*	*60*	*100*	*140*	*180*	*220*	*260*	*300*	*330*
TR=1.25%, DLG=2.5mm	3%/3 mm	98.4	97.2	99.3	97.9	97.6	97.8	97.8	96.9	98
	2%/2 mm	95.1	94	95.5	94.7	94.5	94.1	94.5	91.3	94.8
	1%/1 mm	80	77.3	84.5	78.3	76.8	75.7	82.3	75.7	79.7
TR=1.4%, DLG=2.3 mm	3%/3 mm	99.8	99.6	99.5	99.5	99.2	99.6	99.6	99.3	99.6
	2%/2 mm	99.1	97.8	98.2	96.8	96.9	97.2	96.8	95.9	97.6
	1%/1 mm	90.7	84.9	91.3	85.6	85.3	85.1	88.3	85.3	87.6

GA=gantry angle in the treatment plan (the gantry angle in the QA plan was always 0°); DTA=DTA test; gamma=gamma test.

**Table 3:** Individual QA pass rate with different criteria for each field of one head‐and‐neck plan having the specified transmission ratio and DLG parameters, using the DTA test and the gamma test. The Van Dyk was OFF

*GA DTA*		*15*	*20*	*180*	*230*	*260*	*310*	*345*
TR=1.25%, DLG=2.5mm	3%/3 mm	98.1	97.5	95.2	96.3	97.2	91.1	97.1
	2%/2 mm	91.3	90.0	88.7	86.4	86.1	74.4	85.3
	1%/1 mm	64.2	66.1	58.0	53.3	55.1	44.0	55.0
TR=1.4%, DLG=2.3 mm	3%/3 mm	100	95.0	97.6	98.0	98.7	98.8	98.9
	2%/2 mm	97.3	88.9	93.4	94.1	94.3	94.9	95.0
	1%/1 mm	76.9	64.4	71.4	74.3	72.0	74.3	72.1
*GA Gamma*		*15*	*20*	*180*	*230*	*260*	*310*	*345*
TR=1.25%, DLG=2.5mm	3%/3 mm	99.3	99.5	96.7	98.3	98.4	94.2	98.9
	2%/2 mm	94.6	93.0	90.6	89.4	88.2	77.9	87.2
	1%/1 mm	64.1	66.4	59.0	52.6	55.0	46.1	54.3
TR=1.4%, DLG=2.3 mm	3%/3 mm	100	96.9	99.6	99.1	99.7	100	100
	2%/2 mm	99.5	92.1	96.4	96.8	97.4	98.1	96.7
	1%/1 mm	74.9	65.4	71.7	73.8	71.1	76.2	71.0

GA=gantry angle in the treatment plan; DTA=DTA test; gamma=gamma test.

**Table 4:** Individual QA pass rate with different criteria for each field of a right parietal plan having the specified transmission ratio and DLG parameters in Pinnacle and Eclipse, with Van Dyk ON and OFF. Gamma=gamma test

*Field Index Gamma Van Dyk ON*		*1*	*2*	*3*	*4*	*5*	*6*	*7*	*8*	*9*	*10*
TR=1.24%, DLG=0.4 mm	3%/3 mm	91.5	95.2	96.2	86.9	91.8	95.6	96.1	94.7	97.6	94.2
	2%/2 mm	71.8	73.2	81.7	77.0	73.6	80.3	84.1	81.4	86.6	78.5
	1%/1 mm	42.5	44.6	56.7	53.9	41.6	43.9	52.2	51.8	59.0	46.5
TR=0.26%, DLG=0.3 mm	3%/3 mm	95.1	95.9	96.3	97.7	100	97.3	99.2	99.2	97.8	98.3
	2%/2 mm	84.1	90.1	85.1	79.8	92.4	87.9	92.9	87.6	94.1	89.3
	1%/1 mm	44.5	53.3	45.0	45.4	59.6	54.7	60.0	49.4	62.3	57.6
*Field Index Gamma Van Dyk OFF*		*1*	*2*	*3*	*4*	*5*	*6*	*7*	*8*	*9*	*10*
TR=1.24%, DLG=0.4 mm	3%/3 mm	80.6	86.6	89.7	81.2	76.2	91.2	89.2	82.7	89.9	85.5
	2%/2 mm	59.5	69.3	75.7	71.7	58.4	69.7	78.4	69.0	74.5	69.2
	1%/1 mm	35.4	40.3	48.7	40.8	35.1	39.0	46.2	41.2	46.6	32.0
TR=0.26%, DLG=0.3 mm	3%/3 mm	90.6	90.9	91.4	96.8	96.4	93.7	98.8	95.6	95.6	97.7
	2%/2 mm	76.9	79.8	73.2	76.6	84.4	82.1	86.2	81.1	86.3	85.3
	1%/1 mm	39.3	43.4	38.3	44.5	56.0	47.1	52.1	46.2	54.2	53.7

### E. Comparison of plan quality

**Figure 4:**
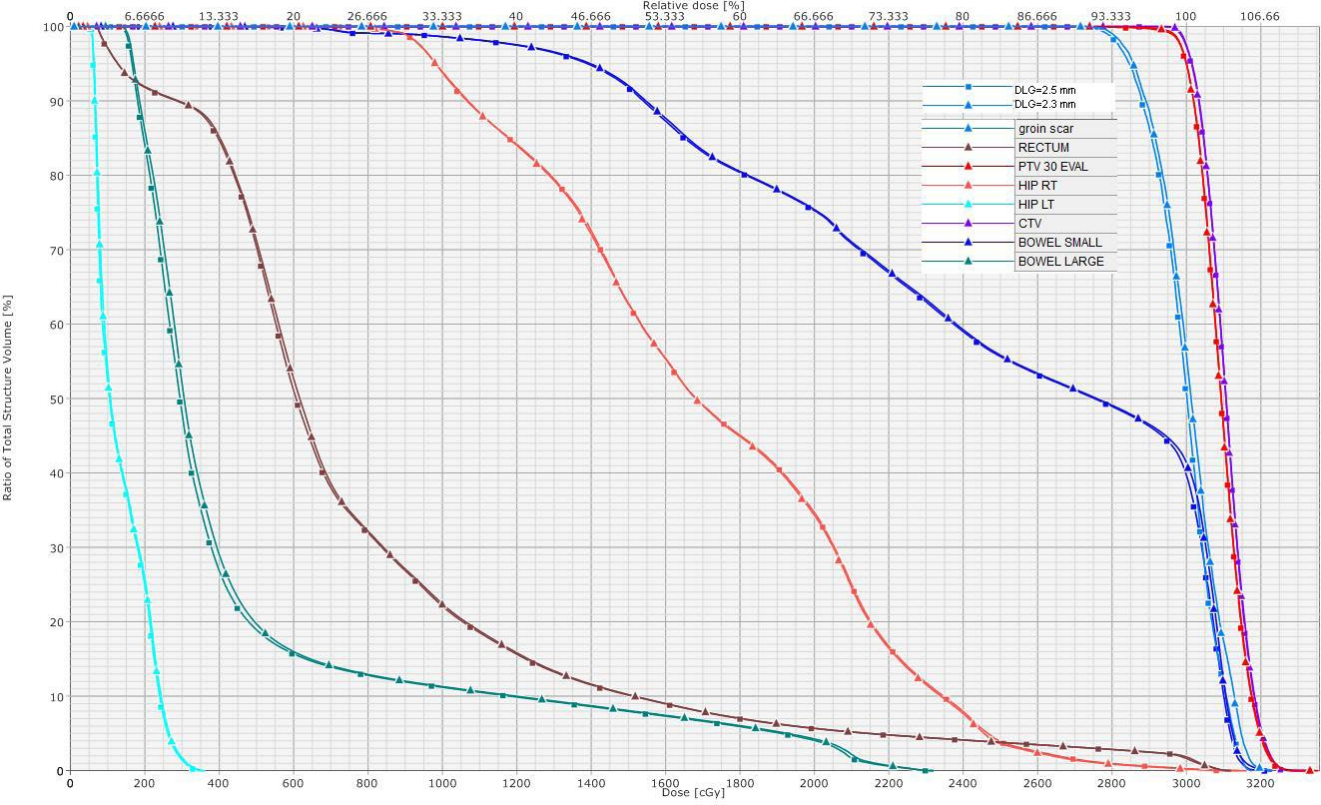
DVHs in pelvis IMRT plans in which the value of DLG was optimized from test fields (in triangle) and typical patient plans (in square).

We investigated the effect of using an optimal DLG on plan quality (i.e., dose volume histograms (DVHs)) of the target and organs at risk. Overall, there was no obvious difference in DVHs generated after using DLGs optimized from patient plans, test fields, or the Pinnacle TPS optimization process. This finding was expected because the inverse planning algorithm optimizes the dose coverage based on the objective constraints. The MLC dosimetric data may affect the dose agreement, but not the dose coverage in the plan. Figure 4 depicts the DVHs of IMRT plans for a pelvis patient. The DVHs of PTV and CTV were almost identical, and hips too. The dose to the groin scar was a little higher, with a DLG of 2.3 mm rather than 2.5 mm, but was balanced by a little higher dose to bowels.

## IV. DISCUSSION

Different approaches to measuring the DLG have been proposed by many authors, and similar results have been obtained.^20^, ^21^ Due to the complexity of the MLC system, using the measured DLG cannot guarantee good agreement of planned and measured doses. Before the dose calculation algorithm's capability in dealing with the complicated system can be improved, one may use an optimal value for DLG instead of the measured DLG. Or, if the measured DLG is used, then an additional parameter may be assigned to compensate for the efforts of the dose calculation algorithm on small fields and the T&G effects.^22^, ^23^

Compared to the optimization scheme using typical patient plans, our approach using test fields provides a view of the capability of the dose calculation algorithm in a wide range of leaf gaps and T&G extensions. In our clinic, we had several failed pelvis IMRT QA plans, mainly due to the large volume of the targets, which had not been used as typical patient plans during commissioning. Furthermore, the value of the DLG optimized for one dose calculation algorithm cannot be used in another algorithm because each algorithm may deal with the MLC system differently. In this work, we found that using a transmission ratio of 1.24% and a DLG of 0.4 mm, optimized by Pinnacle TPS, into Eclipse TPS resulted in a systematically negative difference between the measured and planned doses. This outcome occurs because, with fixed MU delivery, use of higher than the physical value of transmission ratio results in a higher planned dose.

From our test fields, each gap and T&G extension has a corresponding optimal DLG, but any patient plan consists of multiple gaps and T&G extensions. We have to choose the optimal DLG that works in the largest possible distribution region of gaps and T&G extensions in most patient plans, namely, the linac‐specific optimal DLG. Thus, theoretically, a single “optimal” DLG may fail in some special plans, although, practically, we have not had such a case. Because of the critical importance of the delivered dose matching with the planned one, it is valuable to use a plan‐specific optimal DLG for such a specific plan if the linac‐specific optimal DLG does not work. A better solution is to include the result from the test fields into the leaf motion calculator because there is freedom to arrange the leaf positions for a given dose distribution.^24^, ^25^

The mean leaf gap in VMAT is often larger than that in IMRT,^(26)^ so that the dose agreement for VMAT will likely be higher. However, it was also observed that the leaf gaps in VMAT plans could be widely distributed from small to large; another fact is that the aperture size in the longitudinal direction may not be consistently larger in VMAT plans than in IMRT plans. Thus, the QA of VMAT plans could fail. An optimal value for the DLG is still essential for dose agreement. We found that the optimal DLG for IMRT plans also worked for VMAT plans. This finding is not surprising if the same dose calculation algorithm is used for both IMRT and VMAT plans. So far, Varian has not allowed the user to export VMAT MLC files. Therefore, we cannot perform a quantity analysis for T&G extension and gap distributions in VMAT plans.

From the investigated regions of gap and T&G extension (1, 2, 3), the Siemens 160 MLC system has a DLG less than that in the Varian millennium and HD MLCs. This is mainly due to its small triangular T&G from the slightly tilted angle. Particularly as shown in Fig. 2(d), the percentage difference between measured and calculated doses for the Siemens 160 MLC system has a wider range than those in the Varian MLC systems when the gap is less than 2 cm, but becomes similar when the gap is greater than 2 cm. This indicates that small fields should be avoided for the Siemens 160 MLC system in order to have a high dosimetry agreement. The system should work in larger than 2 cm gaps and less than 1 cm T&G extensions for good dose agreement. In Pinnacle TPS with the step‐and‐shoot technique, the MLC segment is commonly set to be at least 2 cm. Thus, our work supports this common choice. Currently, the Eclipse TPS does not have an option to set the minimal size of MLC segments for the step‐and‐shoot technique, but the requirement on MU efficiency in the leaf‐motion calculator can avoid using small MLC segments. Otherwise, individual QA could be a challenge for plans by Eclipse with step‐and‐shoot technique delivered by the Siemens 160 MLC system. The distribution of gaps and T&G extensions in typical patient plans can be used for the analysis, but so far, Varian allows the user to export MLC files from Eclipse for Varian system only.

It is observed that the gain of QA pass rate from optimal DLG is plan‐dependent, but the lower pass rate of the original plan, the higher gain of the new plan. For those plans where the QA pass rates are very high already, for instance 100%, use of the optimal DLG usually keeps the high pass rate, but occasionally decreases 1% or 2% of the pass rate. This is because the optimal DLG was for the general linac, not for each specific plan. It is possible that the original DLG may be more suitable for some plans.

## V. CONCLUSIONS

We used test fields to obtain optimal DLGs for rounded leaf‐end MLC systems. The test fields cover a wide range of leaf gaps and T&G extensions, which could exist in practical patient IMRT/VMAT plans, and are independent of MLC systems and treatment sites. Thus, it is more complete and efficient to use the test fields than to use typical patient plans. Our work indicates that there is no single optimal DLG for all possible plans, but some optimal DLG can work for most plans. For special plans with, for example, narrow gaps and large T&G extensions, a plan‐specific optimal DLG may be necessary to ensure that the dose delivered is as planned. Furthermore, each dose calculation algorithm and its version should be commissioned with its own optimal DLG, especially if the algorithm or the version is different from others in dealing with T&G effect, small fields, and other important parameters. It is recommended that, for best dose agreement, an optimal DLG should be obtained for each linac. Our work also supports a generally known result that having more segments with large gaps and small T&G extensions (i.e., large fields) increases the dose agreement.

## ACKNOWLEDGMENTS

We thank Dr. Cherise Guess for editing the manuscript. The anonymous reviewers are appreciated for their dedicated comments. Part of this work was done at Eastern Health, Newfoundland, Canada. WY thanks David Goodyear, Michael Gerald, and Maria Corsten for fruitful discussions.
